# Aroylhydrazone Cu(II) Complexes in *keto* Form: Structural Characterization and Catalytic Activity towards Cyclohexane Oxidation

**DOI:** 10.3390/molecules21040425

**Published:** 2016-03-29

**Authors:** Manas Sutradhar, Elisabete C. B. A. Alegria, M. Fátima C. Guedes da Silva, Luísa M. D. R. S. Martins, Armando J. L. Pombeiro

**Affiliations:** 1 Centro de Química Estrutural, Instituto Superior Técnico, Universidade de Lisboa, Av. Rovisco Pais, 1049-001 Lisboa, Portugal; ebastos@deq.isel.ipl.pt (E.C.B.A.A.); fatima.guedes@tecnico.ulisboa.pt (M.F.C.G.d.S.); lmartins@deq.isel.ipl.pt (L.M.D.R.S.M.); 2 Chemical Engineering Department, Instituto Superior de Engenharia da Lisboa, Instituto Politécnico de Lisboa, R. Conselheiro Emídio Navarro, 1959-007 Lisboa, Portugal

**Keywords:** *O*,*N*,*O* ligands, Cu(II) complex, X-ray structure, cyclohexane oxidation

## Abstract

The reaction of the Schiff base (3,5-di-tert-butyl-2-hydroxybenzylidene)-2-hydroxybenzohydrazide (H_3_L) with a copper(II) salt of a base of a strong acid, *i.e.*, nitrate, chloride or sulphate, yielded the mononuclear complexes [Cu(H_2_L)(NO_3_)(H_2_O)] (**1**), [Cu(H_2_L)Cl]·2MeOH (**2**) and the binuclear complex [{Cu(H_2_L)}_2_(µ-SO_4_)]·2MeOH (**3**), respectively, with H_2_L^−^ in the *keto* form. Compounds **1**–**3** were characterized by elemental analysis, Infrared (IR) spectroscopy, Electrospray Ionisation Mass Spectrometry (ESI-MS) and single crystal X-ray crystallography. All compounds act as efficient catalysts towards the peroxidative oxidation of cyclohexane to cyclohexyl hydroperoxide, cyclohexanol and cyclohexanone, under mild conditions. In the presence of an acid promoter, overall yields (based on the alkane) up to 25% and a turnover number (TON) of 250 (TOF of 42 h^−1^) after 6 h, were achieved.

## 1. Introduction

Alkanes are highly abundant, cheap and thus very attractive potential substrates for the direct synthesis of added value functionalized products. However, their chemical inertness requires harsh temperatures leading to low conversions, limiting their wide usage as raw materials for atom efficient and selective oxidation processes [1,2,3,4]. Therefore, the search for efficient catalytic systems and single-pot methodologies for the selective oxidation of alkanes under mild conditions and using environmentally benign oxidants remains a serious challenge [5,6,7,8,9].

Like the active centres of some enzymes (e.g., particulate methane monooxygenase, a multi-copper enzyme that catalyzes the hydroxylation of alkanes) [10], transition metal complexes can potentially catalyse the functionalization of the non-activated C-H bonds of hydrocarbons under mild reaction conditions [11]. During last few years, the promising approach of design bio-inspired catalysts has been pursued [11,12,13,14,15] and several multi-copper(II) complexes have been found to exhibit a high catalytic activity in the oxidation of cycloalkanes by hydrogen peroxide to the corresponding alkyl hydroperoxides, alcohols and ketones [1,5,11,16,17,18,19,20,21].

The main goal of our present work is to study the catalytic application of aroylhydrazone Cu(II) complexes towards the peroxidative oxidation of cyclohexane under mild conditions. Aroylhydrazones exhibit *keto-enol* tautomerism in solution, and various modes of coordination are found in their metal complexes. The tautomeric form of the ligand in their metal complexes is highly dependent on the pH of the medium and the nature of metal ions [22,23,24,25]. Metal complexes derived from such ligands are found to be active catalysts in various oxidation reactions, e.g., oxidation of alkanes, alkenes, alcohols, sulfoxidation, carboxylation *etc.* [13,18,22,25,26,27], and our group is involved in this track.

We have recently synthesized [17] trinuclear aroylhydrazone Cu(II) complexes derived from the Schiff base of this study (H_3_L) in the *enol* form and studied their activity as homogeneous catalysts towards the peroxidative oxidation of cyclohexane to the corresponding alcohol and ketone. In this study, we have further extended the work, by synthesizing two mononuclear and one binuclear complexes with the same Schiff base in the *keto* form and assessing their catalytic potential in the same catalytic reaction.

## 2. Results and Discussion

**Scheme 1:**
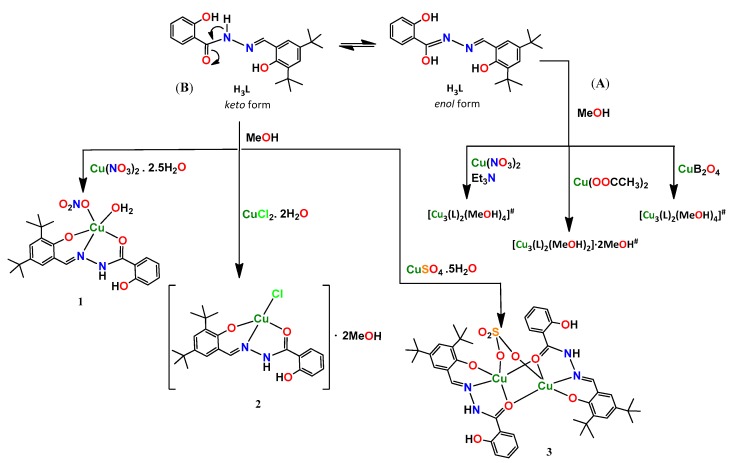
Reaction of H_3_L with different Cu(II) salts. ^#^ See reference [17] for (**A**). This study (**B**).

In a previous work, we reported that the Schiff base (3,5-di-tert-butyl-2-hydroxybenzylidene)-2-hydroxybenzohydrazide (H_3_L) reacts with Cu(NO_3_)_2_·2.5H_2_O in the presence of triethylamine, Cu(OOCCH_3_)_2_·H_2_O or CuB_2_O_4_ (copper metaborate) in methanol producing copper(II) structural isomers with the ligand in the *enol* form [17] (Scheme 1A). Indeed, the Schiff base ligand coordinates to the Cu(II) ions in the *enol* form when the Cu(II) source is a salt of a base of a weak acid, or when the reaction is performed in the presence of a base. In the current work, we have synthesized Cu(II) complexes with the Schiff base in the *keto* form, by using Cu(II) salts with anions derived from strong acids, *viz.* Cu(NO_3_)_2_·2.5H_2_O, CuCl_2_·2H_2_O and CuSO_4_·5H_2_O and performed the reaction with H_3_L in methanol, at room temperature, without the presence of any additional base. The mononuclear compounds [Cu(H_2_L)(NO_2_)(H_2_O)] (**1**), [Cu(H_2_L)Cl]·2MeOH (**2**) and the binuclear complex [{Cu(H_2_L)}_2_(µ-SO_4_)] (**3**) were then isolated (Scheme 1B). In addition, the monoanionic ligand binds the Cu(II) ions in a tridentate κ*O*,*O’*,*N* (in **1** and **2**) or chelating-tridentate fashion (1κ*O*,*O’*,*N*:2κ*O* in **3**), while in the previous situation [17], the fully deprotonated form of the Schiff base (L^−3^) coordinated to the metal in a pentadentate 1κ*O*,*O’*,*N*:2κ*N*’,*O*’’ fashion.

Complexes **1**–**3** have been characterized by elemental analysis, IR spectroscopy, UV-Vis ESI-MS and single crystal X-ray diffraction techniques. In all cases, the IR spectra display the C=O stretching frequency at *ca.* 1613 cm^−1^. The electronic spectral data of **1**–**3** are given in the experimental section. All complexes exhibit intense ligand to metal charge transfer transitions (LMCT) in the range of 225–385 nm and a less intense absorption band due to d–d transitions in the range of 526–660 nm attributable to ^2^B_1g_ → ^2^A_1g_ and ^2^B_1g_ → ^2^E_1g_ transitions, suggesting square-planar to square pyramidal coordination geometries at Cu(II) centre [28,29], which is in agreement with the single crystal structures. The *m/z* values also support the proposed molecular formulation (see Experimental).

### 2.1. Crystal Structures

**Figure 1:**
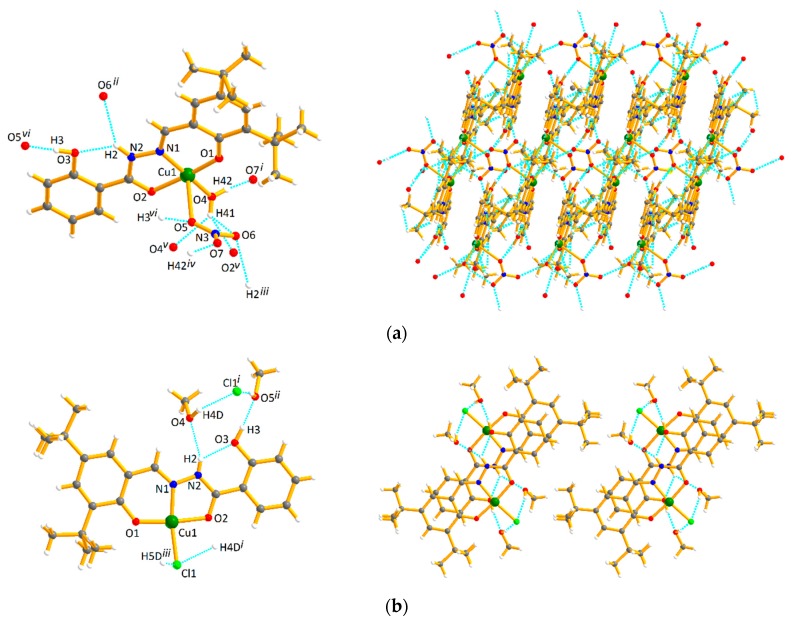

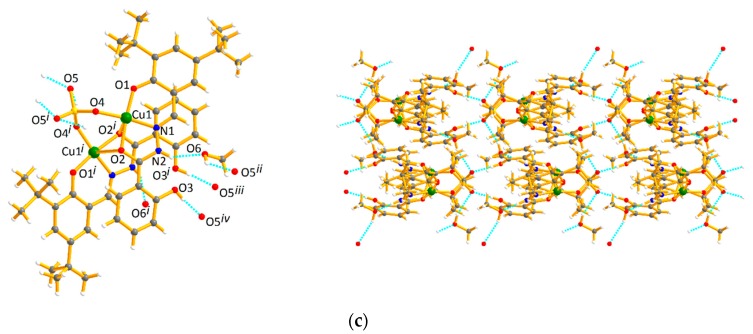
Molecular structures of complexes **1** (**a**); **2** (**b**) and **3** (**c**) with partial atom labeling schemes and respective structural representations of the 3D networks (in **1** and **3**) and dimeric units (in **2**) generated by hydrogen bond interactions (in dashed blue lines). In **1** and **3**, only one of the components of the disordered *terc-*butyl group is shown. Symmetry operations to generate equivalent atoms: **1**: (*i*) −1 + x, y, z; (*ii*) x, −1 + y, z; (*iii*) x, 1 + y, z; (*iv*) 1 + x, y, z; (*v*) 1 − x, 2 − y, 1 − z; (*vi*) 2 − x, 1 − y, 1 − z. **2**: (*i*) −x, −y,1 − z; (*ii*) 1 − x, 1 − y, 1 − z; (*iii*) −1 + x, −1 + y, z. **3**: (*i*) 1 − x, y, ½ − z: (*ii*) x, 1 − y, ½ + z; (*iii*) x, 1 + y, z; (*iv*) 1−x, 1 + y, ½ − z.

At room temperature, methanolic solutions of **1**–**3** yielded X-ray quality single crystals. The crystallographic data (CCDC 1450150-1450152 for **1**–**3** contain the supplementary crystallographic data for this paper. This data can be obtained free of charge from The Cambridge Crystallographic Data Centre via www.ccdc.cam.ac.uk/data_request/cif.) and processing parameters are summarized in Table 1. Selected dimensions and H-bond geometries are presented in Table 2 and Table 3, respectively. The molecular structures of **1**–**3** with atom numbering schemes and illustrations of H-bond interactions are presented in Figure 1.

Compounds **1** and **2** are mononuclear and **3** is dinuclear with the sulphate ligand chelated in a bridging bidentate mode. In complexes **1**–**3,** the H_2_L^−^ ligand bind the metal cations in the tridentate *ONO* (in **1** and **2**) or bridging tridentate µ-*ONO* (in **3**) forms via the phenolate oxygens (O1), the imine nitrogens (N1) and the ketonic oxygens (O2) (Figure 1), the latter binding the two metal cations in **3**. The C-O bond distances involving the ketonic oxygens (O2), in the 1.272–1.265 Å range, confirm the nature of these bonds. The Cu(II) atoms assume square planar (in **2**; τ_4_ = 0.14) [30] or square pyramidal (in **1** and **3**; τ_5_ = 0.11) [31] geometries, the coordination environments being fulfilled with a chloride anion (in **2**), a water and an *O*-nitrate (in **1**) or an *O*-sulphate anion (in **3**). Despite the different geometries and distinct nature of the inorganic anions, the metals are only slightly off [0.059 (**1**), 0.077 (**2**) and 0.065 Å (**3**)] the basal least-square planes. However, in **1** and **2**, the metal cations are engaged in just one 5-membered CuN_2_CO and one 6-membered CuNC_3_O metallacycle; in **3**, two additional metallacycles could be found *viz.* Cu_2_O_3_S and Cu_2_O_2_ and thus, as a result of the bridging nature of both the organic ligand and the sulphate anion, each Cu(II) atom is involved in five cycles. Other distinctive features in the structures of the compounds under study concern the co-planarity of the aromatic rings of the H_2_L^−^ ligands, whose least-square planes make angles of 5.87 (**1**), 4.49 (**2**) and 25.76° (**3**); and the shortest intermolecular Cu···Cu distances which assume values of 5.3831(7) (**1**), 6.639(2) (**2**) and 5.409(2) Å (**3**).

Hydrogen-bond interactions could be found in all compounds (Figure 1 and Table 3), while, in compound **2,** such interactions gather the molecules into dimers as a result of intermolecular contacts between the chloride ligands of two vicinal molecules and four crystallization methanol molecules. In the other cases, the inorganic anions (water and nitrate in **1**; sulphate in **3**) and the crystallization methanol molecules (in **3**) are responsible for extending the structure to the third dimension. In all cases, the phenolic and the amine hydrogens also play a role, the latter donating to the *O*-phenolic (in **3**) or, simultaneously to this type of atom and an *O*-nitrate (in **1**) or an *O*-methanol atom (in **2**).

### 2.2. Catalytic Activity

**Scheme 2:**
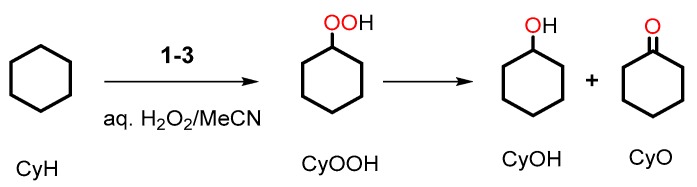
Oxidation of cyclohexane to cyclohexanol and cyclohexanone with CyOOH as primary product.

Peroxidative oxidation of cyclohexane. Following our interest in the Cu-catalyzed oxidative transformation of alkanes under mild conditions [15,32,33,34,35,36], we have chosen cyclohexane (CyH) as a model alkane substrate (Scheme 2) to test the catalytic potential of the synthesized copper compounds **1**–**3**. The catalytic tests were undertaken by reacting at room temperature in MeCN/H_2_O medium, cyclohexane with aqueous hydrogen peroxide (30% aqueous solution) in the presence of **1**–**3**, either in the absence or in the presence of an acid promoter such as the heteroaromatic 2-pyrazynecarboxylic acid (Hpca), trifluoroacetic acid (TFA) or nitric acid.

In order to confirm the formation of cyclohexyl hydroperoxide (CyOOH) as a primary product, we followed a method proposed by Shul’pin [8,37,38,39] by performing, in some cases, the Gas Ghromatography (GC) analysis of the product twice, before and after addition of PPh_3_. The amount of alcohol detected by GC significantly increased after addition of PPh_3_ as a result of the reduction of CyOOH to cyclohexanol (CyOH), with a concomitant decrease of the amount of cyclohexanone (CyO), allowing us to assume the CyOOH as the primary product of this reaction (Scheme 2).

The monocopper(II) **1** and **2** and the dicopper(II) **3** complexes catalyse this reaction very efficiently even in the absence of any additive (Table 4). The dicopper(II) **3** provided the best activity achieving a total yield (cyclohexanone + cyclohexanol) of 18% (relatively to the alkane, Table 4, entry 13) and a turnover number (TON) of 180 (90 per Cu atom), in the absence of any acid co-catalyst, after 6 h reaction. Compounds **1** and **2** exhibit a total yield of 11% and 9% under the same reaction conditions. Further increase of the reaction time from 6 to 24 h increases the total yield of the products from 11% to 29% (for compound **1**, entries 1 and 2, Table 4) and from 18% to 39% (for compound **3**, entries 13 and 14, Table 4), respectively.

For the reactions performed in the presence of **1**–**3** and after 24 h, the reaction mixtures were analyzed before and after its treatment by PPh_3_ (entries 2, 3, 9, 10, 14 and 15, Table 4) and an important difference in the alcohol/ketone molar ratio was observed. This fact points out [8,37,38,39,40] the presence of cyclohexyl hydroperoxide in the reaction mixture even after a long period of time (24 h) as the primary product of cyclohexane oxidation that decomposes to the corresponding alcohol and ketone.

Furthermore, we have found that the presence of a strong acid co-catalyst, such as HNO_3_ (65% solution), 2-pyrazynecarboxylic acid (Hpca), trifluoroacetic acid (TFA), improves the catalytic performance of **1**–**3**. For instance, in the presence of **1**, a growth in total yield of products from *ca*. 11% (entry 1, Table 4) in the absence of any additive to 12%, 14% and 18% in the presence of an excess [n(acid)/n(catalyst) = 25] of HNO_3_ (65% solution) (entry 4, Table 4), Hpca (entry 5, Table 4) and TFA (entry 7, Table 4), respectively.

The promoting effect of an acid co-catalyst was already observed for other Cu-catalysed systems in the oxidative transformation of alkanes [20,35,41,42,43,44,45].

However, it is important to highlight that the levels of activity that have been reached by **1**–**3** (11%, 9% and 18%, respectively), in the absence of any acid promoter, are significant, and this constitutes already a feature of the present catalytic system. Additionally, a high selectivity towards the formation of cyclohexanol and cyclohexanone is exhibited by our system, since no traces of byproducts were detected by Gas Chromatography–Mass Spectrometry (GC-MS) analysis of the final reaction mixtures. Our yield values are higher than that of the industrial process (4% to reach a good selectivity, at *ca.* 150 °C) [2,46].

The activity exhibited by compounds **1**–**3**, in the absence of any additive, is higher than that shown e.g., by other efficient Cu(II) systems such as the complexes bearing azathia macrocycles e.g., [Cu(OTf)_2_(L^3^)] (L^3^ = mixed 14-membered N_2_S_2_ azathia macrocycle) or [Cu(OTf)(L^4^)(H_2_O)](OTf) (L^4^ = nine-membered NS_2_ macrocyclic ligand with a 2-methylpyridyl pendant arm) (overall yield of *ca.* 8%) [41], for the tetranuclear copper(II) complexes [Cu_4_(µ_4_-*O*)(L^1^)Cl_4_] and [Cu_4_(µ_4_-*O*)(L^2^)_2_Cl_4_] [H_2_L^1^ is a macrocyclic ligand resulted from condensation of 2,6-diformyl-4-methylphenol (DFF) and 1,3-bis(aminopropyl)tetramethyldisiloxane); (HL^2^ is a 1:2 condensation product of DFF with trimethylsilyl *p*-aminobenzoate) with an overall yield up to 9%, after 2 h reaction at 50 °C and in the presence of a more concentrated oxidant (H_2_O_2_, 50% aq.) [45] or even for the trinuclear aroylhydrazone Cu(II) complexes derived from Schiff base (3,5-di-tert-butyl-2-hydroxybenzylidene)-2-hydroxybenzohydrazide (H_3_L) in *enol* form [Cu_3_(L)_2_(MeOH)_4_], [Cu_3_(L)_2_(MeOH)_2_]**·**2MeOH and [Cu_3_(L)_2_(MeOH)_4_] (overall yield of *ca.* 5%) [17].

Our **3**/H_2_O_2_ system provides, in the absence of any additive, higher yields than those reported for the arylhydrazones Cu(II) complexes [Cu(H_2_O){(CH_3_)_2_NCHO}(HL)] or [Cu_2_(CH_3_OH)_2_(µ-HL)_2_] [47], prepared by reaction of Cu(NO_3_)_2_·2.5H_2_O with the 3-(2-hydroxy-4-carboxyphenylhydrazone)pentane-2,4-dione, wherein, for example, in the presence of the dicopper [Cu_2_(CH_3_OH)_2_(µ-HL)_2_, a total yield of 14% is reached after 4.5 h of reaction, using a more concentrated oxidant (H_2_O_2_, 50% aq. solution) and performing the reaction at 50 °C while the system under the current study is applied at room temperature.

The obtained yield for our **1**–**3**/H_2_O_2_ systems are comparable to those achieved in the presence of the copper(II) [Cu(H_2_L^1^)(H_2_O)(im)]·3H_2_O and [Cu(H_2_L^2^)(im)_2_]·H_2_O (im = imidazole; H_4_L^1^ = 5-(2-(2-hydroxyphenyl)hydrazono)pyrimidine-2,4,6(1*H*,3*H*,5*H*)-trione; H_4_L^2^ = 2-(2-(2,4,6-trioxotetra-hydropyrimidin-5(2*H*)-ylidene)hydrazinyl)benzenesulfonic acid) complexes (yields up to 21% and TON up to 213) in the peroxidative (with H_2_O_2_) oxidation of cyclohexane after 6 h reaction at room temperature [48].

Experiments in the presence of carbon (2,2,6,6-tetramethylpiperidine-1-oxyl, TEMPO [49,50] and oxygen (Ph_2_NH, diphenylamine) radical traps [51,52] were performed in order to establish the nature of the reaction mechanism. Thus, the addition of TEMPO or Ph_2_NH in a stoichiometric amount relative to cyclohexane leads to a marked inhibition of the products formation (compare entries 18 and 19 with 11, Table 4) leading us to believe that, as in other cases [12,32,35,37,38,53], the reaction proceeds via a radical mechanism, probably involving the formation of an hydroxyl radical (HO•) which can be derived from the reduction of H_2_O_2_ by Cu^I^ (the latter being formed upon oxidation of H_2_O_2_ to HOO• by Cu^II^). The HO• radical conceivably abstracts a hydrogen atom from CyH providing the formation of cyclohexyl radical (Cy•), which reacts with O_2_ (dissolved in reaction medium) to form cyclohexylperoxy radical (CyOO•). The cyclohexylperoxy radical may be reduced by Cu^I^ to the corresponding anion that can be protonated to cyclohexyl hydroperoxide (CyOOH), the primary product of the cyclohexane oxidation, which can undergo a dismutation and produce cyclohexanol, cyclohexanone, and dioxygen. CyOOH can also be formed upon *H*-abstraction of CyOO• from H_2_O_2_ or from the HOO• radical [54].

## 3. Experimental

### 3.1. General Materials and Procedures

All synthetic work was performed in air. The reagents and solvents were obtained from commercial sources and used as received, *i.e.*, without further purification or drying. Three different metal sources *viz.*, Cu(NO_3_)_2_**·**2.5H_2_O, CuCl_2_**·**2H_2_O and CuSO_4_**·**5H_2_O were used for the synthesis of complexes **1**–**3**. C, H, and N elemental analyses were carried out by the Microanalytical Service of the Instituto Superior Técnico. Infrared spectra (4000–400 cm^−1^) were recorded on a Bruker Vertex 70 instrument (Bruker Corporation, Ettlingen, Germany) in KBr pellets; wavenumbers are in cm^−1^. The ^1^H spectra were recorded at room temperature on a Bruker Avance II + 400.13 MHz (UltraShield™ Magnet) spectrometer (Bruker Corporation). The chemical shifts are reported in ppm using tetramethylsilane as the internal reference. The UV-Vis absorption spectra of methanol solutions of **1**–**3** (*ca.* 3 × 10^−5^ M) in 1.00 cm quartz cells were recorded at room temperature on a Lambda 35 UV-Vis spectrophotometer (Perkin-Elmer, Waltham, MA, USA) by scanning the 200–1000 nm region at a rate of 240 nm min^−1^. Mass spectra were run in a Varian 500-MS LC Ion Trap Mass Spectrometer (Varian Inc., Palo Alto, CA, USA) equipped with an electrospray (ESI) ion source. For electrospray ionization, the drying gas and flow rate were optimized according to the particular sample with 35 p.s.i. nebulizer pressure. Scanning was performed from *m/z* 100 to 1200 in methanol solution. The compounds were observed in the positive mode (capillary voltage = 80–105 V).

### 3.2. Typical Procedures for the Catalytic Oxidation of Cyclohexane and Product Analysis

The peroxidative oxidations of cyclohexane with aqueous H_2_O_2_ were performed in round bottom flasks with vigorous stirring, under atmospheric pressure at room temperature, during 6 h. The aroylhydrazone Cu(II) complexes (**1**–**3**) in the presence of an acid cocatalyst such as nitric acid (HNO_3_, 65% solution), pyrazinecarboxylic acid (Hpca)] or trifluoroacetic acid (TFA, in the form of a stock solution in acetonitrile) in MeCN (up to 5.0 mL total volume), were introduced into the reaction mixture. Cyclohexane (5 mmol) was then introduced, and the reaction started when hydrogen peroxide (30% aqueous solution, 10 mmol) was added in one portion. The products analysis was performed as follows: 90 µL of cycloheptanone were added as internal standard together with 10.00 mL of diethyl ether, to extract the substrate and the organic products from the reaction mixture. The obtained mixture was stirred for 10 min; then, an aliquot (1 µL) was taken from the organic phase and analyzed by GC using the internal standard method. At the end of the reaction, before the GC analysis, an excess of triphenylphosphine was added, in order to reduce the formed cyclohexyl hydroperoxide to the corresponding alcohol, and to reduce hydrogen peroxide to water, following a method developed by Shul’pin [8,37,38,39,40,55].

Attribution of peaks was made by comparison with chromatograms of authentic samples. Blank experiments were performed and confirmed that no product of cyclohexane oxidation was obtained unless the metal catalyst was used. GC measurements were carried out using a FISONS Instruments GC 8000 series gas chromatograph (Fisons Instruments), equipped with an Flame Ionization Detector FID detector, a DB-WAX capillary column (length: 30 m; internal diameter: 0.32 mm) (Agilent Technologies, Santa Clara, CA, USA), and He as the carrier gas, and run by the Jasco-Borwin v.1.50 software (Jasco, Tokyo, Japan). The temperature of injection was 240 °C. The column was initially maintained at 100 °C for 1 min, and then it was heated up to 180 °C with steps of 10 °C/min, and held at this temperature for 1 min. The attribution of the observed GC peaks was carried out on the basis of chromatograms acquired on pure cyclohexanol and cyclohexanone samples. Besides that, calibration curves were obtained with known concentrations of samples of pure products and standard.

### 3.3. Preparations

#### 3.3.1. Synthesis of the Pro-Ligand H_3_L

The Schiff base pro-ligand (3,5-di-tert-butyl-2-hydroxybenzylidene)-2-hydroxybenzohydrazide (H_3_L) (Scheme 1) was prepared according to literature by the condensation of salicylhydrazide with 3,5-di-tert-butyl-2-hydroxybenzaldehyde [17].

#### 3.3.2. Syntheses of the Cu(II) Complexes

The aroylhyrazone Cu(II) complexes [Cu(H_2_L)(NO_3_)(H_2_O)] (**1**), [Cu(H_2_L)Cl]·2MeOH (**2**) and [{Cu(H_2_L)}_2_(µ-SO_4_)]·2MeOH (**3**) were prepared by a common method using different Cu(II) salts with anions derived from strong acids; Cu(NO_3_)_2_**·**2.5H_2_O, CuCl_2_**·**2H_2_O and CuSO_4_**·**5H_2_O, respectively. The synthesis of **1** is indicated below, as the general procedure.

##### [Cu(H_2_L)(NO_3_)(H_2_O)] (**1**)

To a 20 mL methanol solution of H_3_L (0.368 g, 1.00 mmol), Cu(NO_3_)_2_**·**2.5H_2_O (0.245 g, 1.05 mmol) was added and the reaction mixture was stirred for 15 min at room temperature. The resultant dark green solution was filtered and the filtrate was kept in open air. After two days, green single crystals suitable for X-ray diffraction analysis were isolated. Yield: 0.372 g (73%, with respect to Cu). Anal. Calcd for C_22_H_29_CuN_3_O_7_ (**1**): C, 51.71; H, 5.72; N, 8.22. Found: C, 51.64; H, 5.69; N, 8.16. IR (KBr; cm^−1^): 3390 and 3212 ν(OH), 2956 ν(NH), 1613 ν(C=O), 1384 ν(NO_3_) and 1198 ν(N–N). UV-Vis _max_ (MeOH, nm (ε, LM^−1^ cm^−1^)): 656 (305), 378 (16,478), 285 (23,434), 237 (32,108). ESI-MS (+): *m/z* = 512 [M + H]^+^ (100%).

##### [Cu(H_2_L)Cl]·2MeOH (**2**)

Yield: 0.4 g (75%, with respect to Cu). Anal. Calcd for C_24_H_35_ClCuN_2_O_5_ (**2**): C, 54.33; H, 6.65; N, 5.28. Found: C, 54.28; H, 6.62; N, 5.23. IR (KBr; cm^−1^): 3436 and 3203 ν(OH), 2958 ν(NH), 1611 ν(C=O), and 1173 ν(N–N). UV-Vis _max_ (MeOH, nm (ε, LM^−1^ cm^−1^)): 526 (338), 380 (18,364), 284 (24,632), 238 (32,328). ESI-MS (+): *m/z* = 467 [(M-2MeOH) + H]^+^ (100%).

##### [{Cu(H_2_L)}_2_(µ-SO_4_)]·2MeOH (**3**)

Yield: 0.394 g (77%, with respect to Cu). Anal. Calcd for C_46_H_62_Cu_2_N_4_O_12_S (**3**): C, 54.05; H, 6.11; N, 5.48. Found: C, 54.0; H, 6.08; N, 5.44. IR (KBr; cm^−1^): 3386 and 3218 ν(OH), 2959 ν(NH), 1614 ν(C=O), and 1147 ν(N–N). UV-Vis _max_ (MeOH, nm (ε, LM^−1^ cm^−1^)): 632 (228), 382 (14,268), 284 (21,436), 234 (30,018). ESI-MS (+): *m/z* = 1023 [M + H]^+^ (100%).

### 3.4. X-ray Measurements

X-ray quality single crystals of complexes **1**–**3** were immersed in cryo-oil, mounted in Nylon loops and measured at a temperature of 296 (**1** and **3**) or 150 K (**2**). Intensity data were collected using a Bruker AXS-KAPPA APEX II PHOTON 100 diffractometer (Bruker AXS Inc., Madison, WI, USA) with graphite monochromated Mo-Kα (λ 0.71073) radiation. Data were collected using omega scans of 0.5° per frame and full sphere of data were obtained. Cell parameters were retrieved using Bruker SMART [56] software and refined using Bruker SAINT [57] on all the observed reflections. Absorption corrections were applied using SADABS [57]. Structures were solved by direct methods by using SIR-97 [58] and refined with SHELXL–2014 [59]. Calculations were performed using the WinGX-Version 2014.01 [60]. The hydrogen atoms attached to carbon atoms were inserted at geometrically calculated positions and included in the refinement using the riding-model approximation; Uiso(H) were defined as 1.2 Ueq of the parent carbon atoms for phenyl residues and 1.5 Ueq of the parent carbon atoms for the methyl groups. The hydrogen atoms of methanol molecules and of the imine groups were located from the final difference Fourier map and the isotropic thermal parameters were set at 1.5 times the average thermal parameters of the belonging oxygen or nitrogen atoms. One of the *terc*-butyl groups in **1** and **3** are disordered over two sites of occupancies 0.47:0.51 (in **1**) and 0.55:0.45 (in **3**) and were refined with the use of PART instructions. Least square refinements with anisotropic thermal motion parameters for all the non-hydrogen atoms and isotropic for the remaining atoms were employed.

## 4. Conclusions

We have successfully synthesized three new Cu(II) complexes (**1**–**3**) derived from the Schiff base (3,5-di-tert-butyl-2-hydroxybenzylidene)-2-hydroxybenzohydrazide (H_3_L) with the coordinated ligand in *keto* form. The nature of the starting Cu(II) salts played a key role for stabilizing the *keto* form over the *enol* one. Compounds **1**–**3** are catalysts for the peroxidative oxidation of cyclohexane to a mixture of cyclohexyl hydroperoxide, cyclohexanol and cyclohexanone in the absence of any additive, in NCMe/H_2_O media and under mild conditions. Cyclohexanol and cyclohexanone are the main products of oxidation (overall yield of 18% for **3**, TON of 180), obtained via formation of cyclohexyl hydrogen peroxide (CyOOH) following a radical mechanism as substantiated by radical trap experiments. Yields may be enhanced by prolonging the reaction time achieving e.g., for **3,** a maximum overall yield of 39% (maximum TON 392) after 24 h, at room temperature and without any additive or by adding an acid co-catalyst (overall yield of 25% for **3**, in the presence of TFA).

## Figures and Tables

**Table 1:** Crystal data and structure refinement details for complexes **1**–**3**.

	1	2	3
Empirical formula	C_22_H_29_Cu∙N_3_O_7_	C_24_H_35_ClCu N_2_O_5_	C_46_H_62_Cu_2_N_4_O_12_S
Formula Weight	511.02	530.53	1022.13
Crystal system	Triclinic	Triclinic	Monoclinic
Space group	*P*-1	*P*-1	*C*2/*c*
Temperature/K	296 (2)	150 (2)	296 (2)
*a*/Å	6.8442 (8)	6.856 (2)	37.902 (19)
*b*/Å	8.8397 (10)	13.166 (5)	10.738 (4)
*c*/Å	20.419 (2)	14.628 (5)	12.920 (6)
α/°	77.915 (4)	77.747 (16)	90
β*/*°	88.361 (4)	86.071 (17)	109.210 (16)
γ/°	83.059 (3)	89.369 (17)	90
*V* (Å^3^)	1199.1 (2)	1287.2 (8)	4965 (4)
*Z*	2	2	4
D_calc_ (g cm^−3^)	1.415	1.369	1.367
µ(Mo Kα) (mm^−1^)	0.957	0.988	0.961
Rfls. collected/unique/observed	8926/4197/3191	33,721/4900/2698	19,819/4647/2866
*R*_int_	0.0401	0.1921	0.0995
Final *R*1 ^a^, *wR*2 ^b^ (*I ≥* 2*σ*)	0.0487, 0.1045	0.0564, 0.0949	0.0487, 0.1106
Goodness-of-fit on *F*^2^	1.061	0.974	1.032

^a^ R *= ∑*||*F_o_*|–|*F_c_*||/*∑*|*F_o_*|; ^b^ wR(F^2^) = [*∑*w(|F_o_|^2^ – |F_c_|^2^)^2^/*∑*w|F_o_|^4^]^½^.

**Table 2:** Selected bond distances (Å) and angles (°) in complexes **1**–**3**.

	1	2	3
Cu1—O1	1.854 (3)	1.870 (3)	1.848 (3)
Cu1—O2	1.967 (3)	1.965 (3)	1.983 (3)
Cu1—O4	1.964 (3)	-	1.914 (3)
Cu1—N1	1.916 (3)	1.917 (4)	1.898 (3)
Cu1—Cl1	-	2.2354 (14)	-
C16—O1			
O1—Cu1—N1	93.11 (12)	92.57 (13)	93.20 (13)
O1—Cu1—O2	173.82 (11)	172.54 (13)	173.34 (11)
O1—Cu1—O4	94.30 (12)	-	95.17 (13)
O1—Cu1—Cl1	-	95.06 (10)	-
O2—Cu1—Cl1	-	92.04 (10)	-
O4—Cu1—O2	91.85 (11)		91.18 (12)
N1—Cu1—O2	80.94 (11)	80.79 (13)	80.92 (13)
N1—Cu1—O4	167.48 (12)		166.71 (13)
N1—Cu1—Cl1		168.18 (11)	

**Table 3:** Hydrogen-bond geometry (Å, °) in complexes **1**–**3** ^a^.

*D*—H···*A*	H···*A*	*D*···*A*	*D*—H···*A*
**1**			
N2—H2···O3	1.96 (9)	2.622 (4)	128 (4)
N2—H2···O6 ^ii^	2.37 (9)	3.130 (4)	141 (8)
O4—H42···O7 ^i^	1.94 (3)	2.797 (4)	166 (9)
O3—H3···O5 ^vi^	1.69 (9)	2.600 (4)	167 (9)
O4—H41···O2 ^v^	2.39 (6)	2.986 (4)	126 (8)
O4—H41···O4 ^v^	2.36 (7)	2.977 (6)	129 (7)
O4—H41···O6	2.52 (8)	2.998 (4)	116 (6)
**2**			
O3—H3···O5 ^ii^	1.74 (2)	2.598 (5)	169 (4)
O4—H4D···Cl1 ^i^	2.52 (2)	3.360 (3)	164 (4)
O5 ^ii^—H5D ^ii^···Cl1 ^i^	2.28 (2)	3.148 (3)	171 (4)
N2—H2···O4	2.26 (5)	2.999 (5)	151 (4)
N2—H2···O3	2.11 (4)	2.643 (5)	123 (4)
**3**			
O6—H6*D*···O5 ^ii^	2.43 (6)	3.166 (5)	139 (8)
N2—H2···O3	2.15 (8)	2.620 (4)	114 (7)
N2—H2···O6	2.06 (8)	2.839 (5)	151 (8)
O3—H3···O5 ^iv^	1.79 (8)	2.620 (4)	177 (10)

^a^ For symmetry codes, see Figure 1.

**Table 4:** Total yield (cyclohexanol and cyclohexanone) with time in the oxidation of cyclohexane by H_2_O_2_ (30% aq. solution) at room temperature in CH_3_CN catalyzed by **1**–**3** ^a^.

Entry	Precatalyst	Acid Co-Catalyst ^b^	Reaction Time (h)	Yield (%) ^c^			Total TON [TOF (h^−1^)] ^d^
				CyOH	CyO	Total	
1	**1**	-	6	10.8	0.6	11.4	114 (19)
2		-	24	27.9	1.5	29.4	294 (12)
3 ^e^		-	24	11.0	8.3	19.3	193 (8)
4		HNO_3_ (25)	6	10.4	0.9	12.3	123 (21)
5		Hpca (25)	6	12.5	1.1	13.6	136 (23)
6		TFA (10)	6	13.4	1.1	14.5	145 (24)
7		TFA (25)	6	17.3	0.9	18.2	182 (30)
8	**2**	-	6	7.8	1.3	9.1	91 (15)
9		-	24	18.7	2.6	21.3	213 (9)
10 ^e^		-	24	6.2	4.9	11.1	111 (5)
11		HNO_3_ (25)	6	13.0	1.6	14.6	146 (24)
12		TFA (25)	6	14.2	2.0	16.2	162 (27)
13	**3**	-	6	16.4	1.6	18.0	180 (30)
14		-	24	37.6	1.6	39.2	392 (16)
15 ^e^		-	24	18.7	3.3	22.0	220 (9)
16		HNO_3_ (25)	6	18.7	1.6	20.3	203 (34)
17		TFA (25)	6	22.9	2.3	25.2	252 (42)
18 ^f^		-	6	1.2	0.4	1.6	16 (3)
19 ^g^		-	6	0.5	0.4	0.9	9 (2)

^a^ Reaction conditions (unless stated otherwise): cyclohexane (5.0 mmol), catalyst precursor **1**–**3** (5 µmol, 0.1 mol % *vs.* substrate), H_2_O_2_ (10.0 mmol), MeCN (up to 5 mL total volume), room temperature; ^b^ n(acid)/n(catalyst); ^c^ moles of products [cyclohexanol (CyOH) + cyclohexanone (CyO)]/100 mol of cyclohexane, determined by GC after treatment with PPh_3_; ^d^ turnover number = moles of products (cyclohexanol + cyclohexanone) per mol of catalyst; TOF = TON per hour (values in brackets); ^e^ GC injection before PPh_3_ addition; ^f^ in the presence of TEMPO (5.0 mmol); ^g^ in the presence of diphenylamine (5.0 mmol).
